# 

**Published:** 1857-07

**Authors:** 


### EDITORIAL.

*To Subscribers.*

The present number begins the last half of the present volume, and in it we inclose bills to all, or nearly all, who stand indebted on our books. A few, who have remitted their subscriptions, or paid our agent, since these bills were made out, will be kind enough to disregard the bill, and look for an acknowledgment of their remittances on the second page of the cover to this and succeeding numbers of the Journal. Those who have not yet paid (and they are many), will do us a great favor by doing so immediately. The amount due from any one individual is small, but the aggregate is large, and to us very important, for the printer wants his pay.

### RECEIPTS ON SUBSCRIPTIONS,

UP TO JULY 1ST, 1857.

### ILLINOIS.

| | | | |
|---|---|---|---|
| Dr. Thos. D. Washburn, | Vol. | 6, | $5.00 |
| " A. J. Crain, - - | " | 5, | 2.00 |
| " D. E. Foote, - - | " | 6, | 6.00 |
| " F. B. Haller, - - | " | 6, | 4.00 |
| " G. W. Philips, - | " | 6, | 2.00 |
| " A. H. Luce, - - | " | 6, | 2.00 |
| " Thos. Hall, - - | " | 6, | 2.00 |
| " Wm. Stapps, - - | " | 6, | 2.00 |
| " Jas. Mahan, - - | " | 6, | 2.00 |
| " A. G. Randall, - | " | 6, | 2.00 |
| Drs. J. G. & Z. H. Whitmire, | | 6, | 2.00 |
| Dr. Wm. McNight, - | " | 4&5, | 4.00 |
| " J. F. Hamilton, - | " | 5&6, | 4.00 |
| " M. B. Baldwin, - | " | 4, 5&6, | 5.00 |
| " W. P Naramore, - | " | 5&6, | 4.00 |
| " C. M. Hamilton, - | " | 6, | 2.00 |
| " Thos. B. Trower, - | " | 6, | 2,00 |
| " A. F. Hand, - - | " | 6, | 4.00 |
| " P. S. Secor, - - | " | 6, | 2.00 |
| " E. Wenger, - - | " | 5&6, | 4.00 |
| " E. Richards, - - | " | 6, | 2.00 |

### INDIANA.

| | | | |
|---|---|---|---|
| Dr. A. B. Buchanan, | Vol. | 5&6 | $4.00 |
| Drs. Coe & Constant, | " | 5, | 2.00 |
| Dr. E. H. Sutton, - - | " | 5, | 2.00 |
| " D. Hutchinson, - | " | 6, | 2.00 |
| " Asa T. Hardy, - | " | 6, | 2.00 |
| " J. M. Logan, - - | " | 4&5, | 5.00 |

### IOWA.

| | | | |
|---|---|---|---|
| Dr. Wm. Watson, - | Vol. | 6, | $2.00 |
| " W. M. Line, - - | " | 6, | 2.00 |

### MICHIGAN.

Drs. Hopkins & Garwood, Vol. 6, $2.00

### WISCONSIN.

Dr. Adolphus Bodenstab, Vol. 6, $2.00
" Treat, - - - Vol. 4, 5&6, $6.00

### KANSAS.

Dr. W. B. Swisher, - - Vol. 6, $2.00

